# Make the most of what you have! Exploring circularity practices adoption in olive milling through the norm activation model

**DOI:** 10.1371/journal.pone.0337025

**Published:** 2026-09-08

**Authors:** Giuseppe Di Vita, Daniela Spina, Angelo Pulvirenti, Laura Giuffrida, Marcella Rizzo, Giacomo Falcone, Mario D'Amico, Raffaele Zanchini

**Affiliations:** 1 Department of Agriculture, Food and Environmental (Di3A), University of Catania, Catania, Italy; 2 Department of Economics and Business (DEI), University of Catania, Catania, Italy; 3 Department of Agriculture, Mediterranean University of Reggio Calabria, Catania, Italy; 4 Department of Agricultural, Forest and Food Sciences (DISAFA), University of Torino, Turin, Italy; Gannon University, UNITED STATES OF AMERICA

## Abstract

The transition toward a circular economy (CE) in agriculture relies not only on technological progress but also on the behavioural readiness of millers to embrace sustainable practices. This study employs the Norm Activation Model (NAM) to examine how psychological and structural factors shape CE practices adoption among olive oil mill business owners in Sicily. Drawing on survey data, we conducted a Partial Least Squares Structural Equation Modeling (PLS-SEM) analysis using actual behaviour as dependent variable. The results indicate that Awareness of Consequences and Ascription of Responsibility significantly strengthen Personal Norms, emphasizing their key role in fostering moral engagement with environmental sustainability. Yet, Personal Norms alone do not directly lead to CE practices adoption, highlighting a persistent attitude–behaviour gap typical of traditional farming contexts. Notably, Awareness of Consequences exerts a direct effect on observed behaviour while Personal Norms do not, suggesting that NAM may require adaptation when applied to small, family-run agri-food firms in which the owner-manager translates the cognitive recognition of environmental impacts directly into firm-level decisions. Conversely, education, experience, enterprise size, and milling type are found to be significant drivers of CE participation, indicating that moral motivation must be complemented by suitable organizational and contextual support. The findings provide novel empirical evidence that the CE transition in agri-food systems results from the relation between individual moral activation and organizational capacity. Policy measures should therefore combine behavioural and structural approaches, through education, technical assistance, and targeted incentives, to encourage CE innovation and the possible valorization of olive oil by-products.

## 1. Introduction

The shift toward a circular economy (CE) has become a focal point for multiple industries seeking to improve both resource efficiency and sustainability [1,2]. This transformation is mainly encouraged by EU-level strategies, including the 2015 EU Action Plan for the Circular Economy, which promotes a systemic move away from the linear “take–make–dispose” model toward one centered on reuse and waste minimization [3,4].

The literature offers multiple empirical frameworks addressing how farmers, producers, and processors decide to implement CE business models within food value chains [5–7]. Recent works highlight that adoption behaviours are influenced by a combination of socio-economic, environmental, and institutional drivers, where financial incentives [8], supportive regulation [9], and technological progress [10] play essential roles in facilitating CE engagement.

This trend has progressively influenced multiple sectors, including the agri-food industry [11], and more recently, the olive oil milling sector [12,13]. Olive oil production represents a cornerstone of Mediterranean agriculture, particularly in Italy, where it contributes substantially to the economy, rural employment, and environmental sustainability [13,14]. Italy’s olive oil sector generates approximately €3.3 billion in turnover, and Italy is the second-largest olive oil producer in the world after Spain [15]. Moreover, the olive oil market is growing; indeed, the demand is rising for healthy food products and the growing global awareness of the Mediterranean diet as well. In this context, olive oil plays a prominent role as part of the Mediterranean diet [16,17].

However, olive oil mills also generate substantial by-products, including olive pomace, wastewater, and husks. If not properly managed, these waste materials can contribute to environmental degradation [18]. The traditional extraction process, often resource-intensive and waste-heavy, raises major concerns regarding its environmental footprint and overall sustainability [19]. To address these issues, CE principles promote waste valorization, resource recovery, and the implementation of closed-loop production systems [20].

A growing body of literature has demonstrated the economic and environmental benefits of CE approaches in olive oil production, underlining the importance of technological innovation, supportive policy frameworks, and stakeholder collaboration in driving this transition [21–23]. Nevertheless, the use of CE habits in olive milling remains uneven, influenced by cognitive, social, and institutional factors. Understanding the adoption of circularity among olive mill operators is therefore essential to identifying the drivers and barriers to CE practices. In particular, examining prosocial and environmentally responsible behaviours is crucial, as mill operators may be more inclined to adopt sustainable practices when they feel a personal moral obligation to act responsibly. More specifically, while existing studies on CE in olive oil production have mainly addressed technological innovations, economic feasibility and policy instruments [12,13,21,24,25], comparatively little is known about the psychological and moral antecedents that shape entrepreneurs’ willingness to engage with circular practices [26,27]. This research gap is particularly relevant in traditional, family-run agribusiness contexts such as Sicilian olive milling, where decisions are typically taken by individual entrepreneurs whose values, perceived responsibility and moral obligations strongly mediate their response to external incentives [28,29]. Focusing on personal moral obligation does not deny the relevance of structural drivers, but addresses an underexplored mechanism that helps explain why, even in the presence of supportive policies and technologies, CE adoption among olive millers remains uneven.

Accordingly, this research adopts the Norm Activation Model (NAM) as its conceptual foundation to examine the extent to which personal norms, awareness of consequences, and ascription of responsibility shape the adoption of CE practices within olive oil milling.

Within this context, the study conducts a comprehensive analysis of circularity in Sicilian olive mills, since Sicily, along with Apulia and Calabria, is a key region in Italian extra virgin olive oil production and generates substantial quantities of by-products [30]. The results yield valuable information into the psychological and contextual factors shaping the transition toward CE practices, offering guidance for policymakers and industry stakeholders aiming to foster a more sustainable olive oil sector.

The contribution of this paper is twofold. First, it provides original empirical evidence on how psychological constructs of NAM operate in a setting – olive oil milling in southern Italy – that has so far been examined almost exclusively through technological or economic lenses, despite being a paradigmatic case of agri-food sector with high environmental externalities and slow CE uptake. Second, the paper integrates the psychological core of NAM with firm-level structural variables (size, type of milling activity, experience) within a single PLS-SEM specification, thus jointly testing the moral and the organizational antecedents of CE adoption. This dual specification responds to recent calls in the literature to overcome the dichotomy between psychological and structural explanations of pro-environmental behaviour in agri-food entrepreneurs [29,31–33] and allows the identification of specific levers – such as the type of milling activity – that can guide targeted policy interventions in the olive oil supply chain.

## 2. Conceptual background: Objective and hypotheses

This study explores prosocial and environmentally responsible behaviour among olive oil mill operators to understand their engagement with CE business models. To achieve this, we adopt the Norm Activation Model (NAM) as theoretical framework. Originally developed by Schwartz [34], NAM explains pro-environmental behaviour based on three key constructs as follow: personal norms, awareness of consequences (AC), and ascription of responsibility (AR). Individuals are more likely to adopt sustainable practices when they are conscious of the ecological implications of their acts and feel morally responsible to act [35].

In the framework of circular economy, NAM provides valuable lens to investigate the behavioural motivations behind CE adoption. While economic and technological incentives are important, individual values and moral obligations often play a decisive role [26,36]. The model is employed to examine how these internal motivations influence olive millers’ decisions related to waste valorization, resource efficiency, and sustainability initiatives.

The application of NAM to business operators in agri-food systems requires explicit conceptual justification, since the model was originally developed by Schwartz [34] to explain altruistic helping behaviour and subsequently extended mainly to consumer pro-environmental contexts [26,27]. The choice of NAM in our setting rests on three considerations. First, Sicilian olive milling – and more generally agro-food industry – is dominated by small and family-run firms in which the operator is also the owner-manager [30,37,38]: in such contexts the boundary between the individual and the firm-level decision-maker is blurred, so that operators’ personal values, perceived responsibility and moral activation translate quite directly into firm-level pro-environmental decisions. Second, recent studies have successfully applied NAM, alone or in combination with other behavioural frameworks, to farmers and agri-food entrepreneurs facing pro-environmental decisions [29,35,39,40], as well as to other non-consumer settings such as sustainable transport choices [41] and hospitality operators’ environmentally responsible practices [42], supporting its empirical validity beyond the consumer domain. Third, we adopt NAM as the theoretical lens to capture the moral component of decision-making, while complementing it with socio-demographic and firm-level structural variables (H3 and H4) that account for organizational and contextual conditions [31–33]. At the same time, NAM is not designed to capture market dynamics, regulatory frameworks or supply-chain governance [25], which therefore remain promising directions for future research.

Building on this rationale and given that NAM has been widely applied to behaviours such as waste reduction, energy conservation and sustainable agriculture [26], the study first investigates whether NAM can effectively explain pro-environmental millers’ behaviours regarding CE implementation (H1).

Moreover, existing literature highlights that personal norms are shaped by constructs such as awareness of consequences, ascription of responsibility, and social norms, that can influence individuals’ moral engagement with sustainability issues [27]. These constructs could reinforce or hinder the development of strong personal norms, affecting the likelihood of CE adoption. To address this, the study verifies the hypothesis whether personal norms are influenced by other NAM constructs and if AR is affected by AC in shaping millers’ decision-making processes (H2).

Additionally, pro-environmental behaviour in agriculture refers to actions that minimize environmental impact while maintaining productivity. This includes sustainable soil management, water conservation, reduced pesticide use, biodiversity protection, and CE practices [19]. Existing literature highlight how socio-demographic characteristics can influence farmers’ pro-environmental behaviour [28,35]. Factors such as age, education level, income [39,43,44] have been shown to influence behaviour. From this derived the third hypothesis that aims at verifying if socio-demographic characteristics of farmers affect the observed behaviour (H3).

Several firm characteristics can influence farmers’ behaviour toward adopting CE practices. These characteristics determine their capacity, motivation, and willingness to integrate sustainable and resource-efficient strategies into their operations [45–47]. Within this context it has been widely demonstrated that farm size, experience, and access to relevant information influence the formation of their attitudes and their willingness to adopt sustainable practices [48–50].

In light of these last premises, the study aims to examine which firm characteristics influence millers’ behaviours toward adopting CE practices (H4).

**Fig 1:**
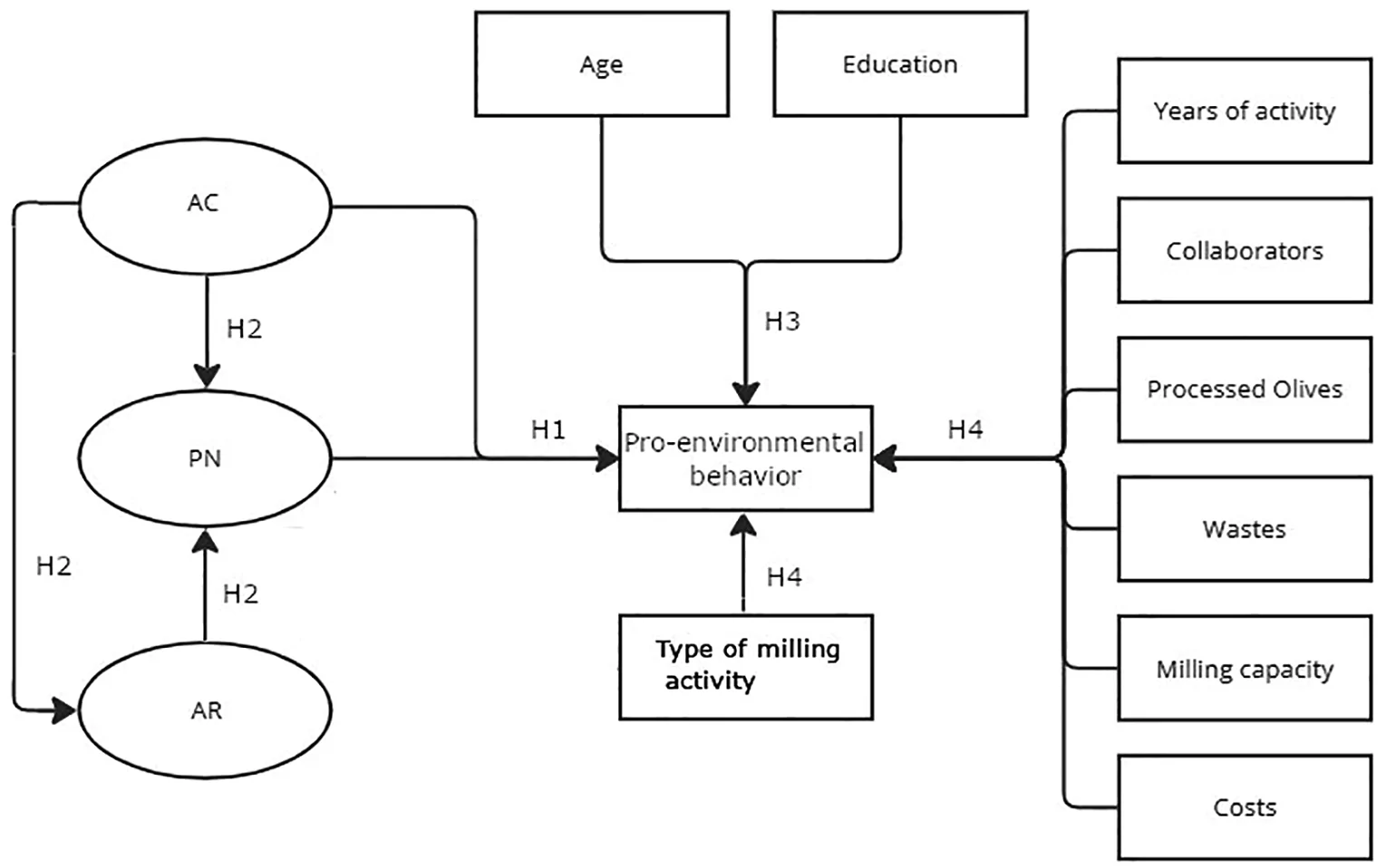
Relations explored in the structural model combining NAM with explorative items. The model tests the relationships between awareness of consequences (AC), ascription of responsibility (AR), and personal norms (PN) in influencing pro-environmental behaviour (H1–H4). Socio-demographic variables (age, education) and firm-level characteristics (years of activity, collaborators, processed olives, wastes, milling capacity, and costs) are included as influencing factors, together with the type of milling activity.

The findings offer insights into how policy measures, educational initiatives, and industry collaboration can promote a more sustainable and CE approach to olive milling in Sicily and beyond. The relations and hypotheses are depicted in Fig 1.

Based on the theoretical foundations discussed above, the proposed hypotheses can be summarized as follows:

**H1**: The NAM effectively explains business operators’ pro-environmental behavior regarding Circular Economy implementation.

**H2**: Personal norms are positively influenced by other core NAM constructs, and Ascription of Responsibility is significantly affected by Awareness of Consequences.

**H3**: The socio-demographic characteristics of business operators significantly affect their pro-environmental behavior

**H4**: Specific firm-level characteristics significantly influence operators’ behaviors toward Circular Economy practices

## 3. Methodology

### 3.1. Data collection

Data were collected through structured interviews conducted by professionally trained officers from a specialised agency. The survey employed the CAWI (Computer-Assisted Web Interview) method, ensuring a systematic and efficient approach to data gathering. Data collection took place between July and October 2024. Prior to the interview, a preliminary telephone contact was made to provide a detailed explanation of the initiative, request authorisation, and confirm the availability of participants specifically the business owners. Once the respondents had agreed to participate, the questionnaire was briefly explained before the email was sent out to minimize the margin of error during the interviews. Business owners that agreed to take part in the CAWI interview subsequently received an email invitation containing a unique and personalised link to access the survey developed as online questionnaire. The link also provided instructions on how to complete the questionnaire and the option to contact the agency if respondents needed assistance. All data collection procedures adhered to established ethical standards for social science research and were conducted in accordance with the principles of the Declaration of Helsinki. Participating olive oil millers were fully informed about the purpose and scope of the study, the procedures involved, the section and the structure of the survey and the anonymous and aggregated treatment of their data. They were also clearly informed of their right to withdraw from the study at any time without any consequences being the data required solely for research purposes. All participants were adults and provided written informed consent prior to participation, thereby ensuring voluntary participation, as well as the confidentiality and anonymity of the respondents throughout the data collection and analysis phases.

The survey was carefully designed to capture a comprehensive set of data and was divided into three main sections. The first section focused on socio-demographic characteristics of the business owners, including variables such as age, work experience, and education level. Additionally, it collected general information about companies, such as the number of employees, the presence of shareholders, and detailed information on the materials used and generated at different stages of the production process.

The second section explored the structural and productive aspects of the mills, including company size, mill type, and olive production capacity. The total volume of olive processed reflect the scale of production activities, and was therefore used as an indirect proxy for company size, consistent with previous empirical studies in agricultural economics [18]. The type of milling activity was classified into two categories: Own-olive milling, referring to mills processing the operator's own olives, and Contract milling, referring to mills providing milling services for olives supplied by external producers. This part was designed to capture the heterogeneity among businesses in terms of their operational characteristics, production capacity, and processing practices.

The third section assessed the adoption of CE practices within the mills. Business owners were asked to evaluate various elements related to sustainability and resource efficiency using validated scales based on the NAM theoretical frameworks. This part of the questionnaire sought to measure the extent to which CE practices were integrated into business operations, providing insights into the level of environmental commitment and innovation within the sector.

In total, 112 valid responses were retained after a thorough data cleaning process conducted by a specialized agency, ensuring the quality and reliability of the dataset. The interview refusal rate was around 5%. The sample size was considered adequate for studies on entrepreneurs, as the value was in line with recent literature evaluating farmers’ behaviour [31,51] where data collection can be difficult because farmers are often unwilling to be interviewed [52]. Given the number of olive oil millers in Sicily, 616 according to the latest official available data from the Sicily Province [53], the margin of error for a sample size of 112 was calculated using the Raosoft online tool [54] to be 7.04, which is consistent with the current literature on farmers’ behaviour [55]. Finally, in this specific context, the sample dimension represents 18.18% of the total population suggesting the high representativeness of the data. To improve the capability of the Norm Activation Model (NAM) in describing millers’ behaviour, the analysis also incorporated socio-demographic and structural characteristics of both the producers and their firms as predictors of pro-environmental behaviour, as detailed in Table 1. Moreover, the table includes the codes used to quantify the information carried by the categorical socio-demographics and firms related variables allowing a better understanding of the results. Including these exploratory variables as predictors alongside validated psychometric scales is consistent with previous research combining the role of psychometric constructs with individual and structural characteristics [32,56,57]. This strategy provides a more comprehensive representation suggesting that structural and contextual factors can meaningfully be integrated to link individual psychological traits with structural and contextual factors in explaining individual decision-making [31,32].

**Table 1:** Descriptive outcomes of the olive oil millers (n = 112).

Variables	Categories	Codes	Absolute frequency	Relative frequency	Mean (SD)
Age	Age	Continuous	N.A.	N.A.	54.44 (14.89)
Education	Up to Middle school	1	22	19.64	N.A.
	High school	2	58	51.79	N.A.
	University degree	3	32	28.57	N.A.
External collaborators	No	1	22	19.64	N.A.
	Yes	2	90	80.36	N.A.
Years of activity		Continuous	N.A.	N.A.	25.19 (15.49)
Olive processed (t)		Continuous	N.A.	N.A.	3242.09 (4687.84)
Type of milling activity	Own-olive milling (mills processing the operator's own olives	1	56	50.00	N.A.
	Contract milling (external olive mill)	2	56	50.00	N.A.
Wastes (mc)		Continuous	N.A.	N.A.	949.05 (1440.97)
Milling capacity (100 kg/h)		Continuous	N.A.	N.A.	124.96 (519.86)
Costs (€/100 kg)		Continuous	N.A.	N.A.	16.03 (2.90)

### 3.2. The norm activation model

Concerning the part of the survey related to the validated Norm Activation Model (NAM), the items measuring Awareness of Consequences (AC), Ascription of Responsibility (AR), and Personal Norms (PN) were adapted from the validated NAM scale developed by Rezaei et al. [58]. This choice was motivated by the theoretical foundations of NAM, which is specifically designed to explain pro-social and pro-environmental behaviours driven by moral obligation rather than economic incentives [34,59].

The scale proposed by Rezaei et al. [58] offers a contextually appropriate operationalization of NAM constructs in sustainability-related decision-making, making it particularly suitable for adaptation to entrepreneurial and organizational contexts where environmental practices are voluntary, responsibility-based, and potentially costly. Accordingly, the items were contextualized to explicitly refer to pro-environmental behaviour while preserving their original theoretical meaning, in line with established applications of NAM in environmental and sustainability research, see Table 2. These constructs were investigated by developing Likert scales ranging from 1 = “totally disagree” to 5 = “totally agree”.

**Table 2:** List of constructs and items derived from NAM.

Constructs	Items	Mean (SD)
PN1	I feel a sense of moral duty to adopt circular economy practices in my olive mill.	4.51 (0.62)
PN2	Not engaging in environmentally harmful practices makes me feel like a more responsible miller.	4.58 (0.61)
PN3	I feel a moral duty to embrace pro-circularity behaviours in my olive oil processing.	4.45 (0.72)
AR1	I believe I am partly responsible for any negative environmental impacts caused by my mill.	3.56 (0.73)
AR2	I acknowledge that my olive oil processing methods may contribute to environmental degradation, and I am responsible for addressing it.	3.88 (0.76)
AR3	I think all olive millers share the responsibility to adopt practices that protect the environment.	3.96 (0.87)
AR4	I feel myself as responsible to take action and contribute to environmental protection in my olive oil production.	4.37 (0.72)
AC1	Adopting circular economy practices in my mill reduces waste and energy consumption.	4.23 (0.72)
AC2	Implementing circular economy practices helps preserve biodiversity.	4.27 (0.80)
AC3	Current practices in my mill may contribute to environmental pollution.	3.96 (0.97)
AC4	Circular economy practices can help improve air quality.	4.18 (0.65)
AC5	Circular economy practices can reduce soil and water pollution.	4.30 (0.68)
AC6	Circular economy practices can have a positive impact on human health.	4.13 (0.69)

In the analytical phase, the variables or items were assessed through the measurement model and included as predictors in the structural model. The observed behaviour served as the dependent variable and was developed as a binary (dummy) variable, coded as 1 if entrepreneurs reported using at least one CE technique in their companies related to different solid and liquid by-products pathways. Behaviour was treated as a dummy variable because, given the population of olive oil millers in Sicily and the limited sample size that could be obtained considering the difficulties in collecting primary data from business owners [52], a more detailed specification would likely have increased model complexity and reduced coefficients stability. Specifically, modelling behaviours as separate variables could have resulted in categories with few or no observations. In regression analysis, such sparse data can produce unstable estimates and increase the risk of overfitting [60].

A list of potential CE practices was presented to olive oil millers, related to the possible pathways that by-products from olive oil extraction can take. In particular olive oil solid wastes and mill wastewaters were both considered.

A list of potential Circular Economy (CE) practices was presented to olive oil millers, focusing on the alternative pathways through which by-products generated during olive oil extraction could be valorized. In particular, the analysis considered both olive mill solid wastes (e.g., pomace and pruning residues) and olive mill wastewater.

Specifically, the set of CE practices included in the analysis and presented to business owners in the survey was identified through a review of the recent literature on circular economy approaches in the olive oil sector [12]. The selection criterion was based on the effective possibility for millers to directly adopt these practices within their production activities. Accordingly, the following pathways were considered: biofuel production; reuse of pruning residues for regenerative agriculture; use of olive mill wastewater for soil treatments or for the extraction of high-value bioactive compounds; and biofertilizer production.

The adapted Italian version of the NAM scale was subsequently pilot tested with a small sample of eight entrepreneurs drawn from the target population. The pilot test aimed was developed to assess item clarity and response variability requested for an effective data collection improving response reliability rather than scale validation. Indeed, considering the population of olive oil millers and the difficult engagement in pre-testing experiences this solution was considered consistent with the agricultural context and with the aim of the paper. The pilot test was conducted in collaboration between the university staff and the specialized agency enrolled for data collection. Feedback from millers indicated that the items were clear and relevant for the milling context; only minor linguistic adjustments were required, and no items were removed. This phase was also important for understanding the cognitive effort required of business owners in completing the questionnaire. The feedback received was positive, as the entire interview took no more than 10 minutes, and respondents confirmed that their attention span remained sufficiently high throughout the interview.

### 3.3. Data analysis

To address the research hypotheses developed for this study, Partial Least Squares Structural Equation Modeling (PLS-SEM) was deemed as the most suitable analytical approach. This approach is extensively employed in Social Sciences [36,61], PLS-SEM is particularly advantageous for analyzing complex relationships among variables and for identifying latent constructs in social phenomena [62]. PLS-SEM was deemed appropriate for data analysis also considering the small population of millers. Indeed, the model is particularly suitable to formalize relations in small population analysis [63]. This approach is especially effective when using validated scales based on well-established theoretical frameworks like the one used in this work namely Norm Activation Model (NAM) [58]. Considering that PLS-SEM is a distribution-free method, p-values and standard errors cannot be obtained for the structural model’s parameters. Therefore, bootstrap procedure was employed to estimate the standard errors and thus the p-values of the model’s parameters [64]. However, the literature suggests that the standard errors should be interpreted with caution since the value is affected by the number of replications [65]. To reduce the impact of this problem, the number of replications was chosen based on what is indicated in the literature, i.e., 5000 [51,66,67].

In comparison to covariance-based structural equation modeling (CB-SEM), PLS-SEM can yield results with higher reliability when the model includes multiple constructs and indicators [42]. It is also more robust in cases of little sample sizes [63,64] and does not rely on strict distributional assumptions, such as normality of the error term [64,68]. This is particularly important because maximum likelihood estimators in CB-SEM may produce biased results under non-normal conditions [69]. Hence, the PLS-SEM can be preferred if complex constructs are included in the survey, that have multiple items, and smaller samples are collected due to its higher robustness and statistical power. Moreover, it aims to minimize unexplained variance and maximize R² values [70].

PLS-SEM operates through a series of partial regressions and includes two main components: the measurement and structural models [63,71]. The estimation algorithm consists of three stages: (1) iterative scoring of latent variables, (2) individuation of measurement model parameters, and (3) estimation of the parameters related to the structural model, also referred to as path coefficients [64]. The measurement model establishes the relationships between latent constructs and their observed indicators, whereas the structural model assesses the relationships among latent variables within a multivariate regression framework, where variables may serve as either predictors (exogenous) or outcomes (endogenous) [64].

Latent constructs in the model are defined as linear combinations of the observed indicators and are employed for predictive analyses [70]. To assess model fit, several quality criteria were applied since this issue is complicated in PLS-SEM and still not fully defined [64]. Indeed, a comprehensive measure of goodness of fit and many threshold values are still considered very tentative [63].

The indexes employed are related to each other, therefore, firstly to compute the average redundancy, the average communality was obtained. Redundancy assesses the amount of variance in the indicators or items explained by the exogenous latent variables used to predict endogenous variables. The average R² of the model was obtained and was considered adequate with values greater than 0.20 since in Social Sciences are generally considered acceptable [63,70,72]. The results of these indexes were compared with recent literature to understand the validity of the results.

The reliability of the constructs in term of internal consistency has been evaluated running Cronbach’s Alpha and Rho_A, both of which should exceed 0.60. Cronbach’s Alpha values should be allocated within a range from 0.7 or 0.6 in exploratory research to 0.95. Lower values are related to low internal consistency of the construct and higher values suggest undesirable response patterns triggering inflated correlation among error terms [63]. Moreover, Rho_A is combined with Cronbach’s index since it is considered one of the most important and consistent measures of reliability in structural modelling [73].

The Average Variance Extracted (AVE) was employed to evaluate convergent validity of the constructs considering the value of 0.5 as the minimum one acceptable. Indeed, only constructs that met these thresholds were retained for the structural model. This indicator represents the variance explained of the items from a specific construct and is computed by obtaining the mean value of the square of the loadings [63].

The standardized loadings were examined to ensure robustness, and variables with loadings below 0.40 were not included [66,74]. Moreover, all the loadings from the NAM were included in the measurement model since their value exceeded 0.5. Indeed, items related to loadings below this threshold should be removed from the analysis if no valid justifications are provided [63,75].

Multicollinearity represent an important limitation since it could affect and inflate standard errors, distort path coefficients, and reduce model reliability. Therefore, this issue was assessed with the Variance Inflation Factor (VIF) to guarantee the robustness of the estimates using a threshold value of 5 [51,76]. Values within 3 and 5 suggest that collinearity could affect the model but the problem is considered uncritical [75]. In this way, although multicollinearity is not totally excluded, the effects on standard errors and, consequently, on the quality of the estimates are considered tolerable [63].

Finally, the explanatory power of the structural model was evaluated using the Average R² value for the overall model assessment and the individual R² values for each structural equation. The obtained values were assessed and benchmarked against thresholds reported in recent literature. R² should also be considered with caution being the acceptability threshold affected by the context where the analysis is conducted and in some cases values below 0.1 are still considered satisfactory [63].Conversely, high values of R² exceeding 0.9 are considered problematic as well indicating that the model overfits the data [75]. The value obtained was considered adequate in this context being a social field of research and the total population of millers equal to 616.

The analyses were conducted with STATA 17 using the “plssem” package [64].

## 4. Results and discussion

The questionnaire responses outline the characteristics of the olive oil processing industry in Sicily. The sector is largely composed of small, family-run mills and marginal enterprises, with production levels that barely exceed self-consumption or contribute only marginally to overall company income. Several barriers still hinder the implementation of closed-loop systems for reusing vegetation water.

The results obtained through Partial Least Squares Structural Equation Modeling (PLS-SEM), applying the Norm Activation Model (NAM), provide insights into the relationships between personal norms (PN), ascription of responsibility (AR), awareness of consequences (AC), and observed behaviour (Table 2).

The standardized loadings reported in Table 3 meet the acceptability threshold suggested by Hair et al. [66], as all values exceed 0.4. These coefficients reflect the strength of the correlations between items within each construct.

**Table 3:** Measurement model outcomes: factor loadings and goodness of fit stats.

Items\Construct	Personal Norms	Awareness of Consequences	Ascription of Responsibility
PN1	0.835		
PN2	0.783		
PN3	0.874		
AC1		0.815	
AC2		0.532	
AC3		0.651	
AC4		0.951	
AC5		0.622	
AC6		0.879	
AR1			0.643
AR2			0.595
AR3			0.765
AR4			0.816
Cronbach alpha	0.775	0.847	0.675
Rho_A	0.782	0.902	0.717
AVE	0.691	0.573	0.505

Cronbach’s alpha and Rho_A scores for all the constructs are above 0.6, indicating acceptable internal consistency and supporting the adoption of these set of variables or constructs in the structural model. Convergent validity was also evaluated: the Average Variance Extracted (AVE) values for all constructs meet the indicated threshold exceeding the 0.5 score and confirming that each construct can be considered reliable and used to examine its influence on millers’ behaviour [63].

Concerning the goodness of fit for the model, the average communality was 0.762 and the average redundancy 0.246 indicating a good amount of explained variance and predictive capability being the results in line with other studies [77,78]. Finally, the average R² is 0.322, indicating that the explanatory capacity of the model is sufficient [63] and in line with current literature [78,79]. The Average R² is computed by considering all endogenous latent variables included in the model, providing an overall measure of explanatory power that differs from the R² values associated with individual regressions within the structural model [64].

The structural model was performed after checking the measurement model’s goodness of fit. Collinearity was analysed by means of VIF analysis for all constructs and variables included in the final structural model. VIF analysis results are included in Table 4 indicating that the variables are adequate to be employed in the structural model since no value is higher than 5. For the variables relating to “Type of milling activity” and “Wastes,” the VIF values are slightly below the threshold of 5 and are therefore considered sufficiently reliable to be included in the model, also in view of the interesting theoretical implications. It is possible that these relatively high values are due to the technical characteristics of the olive sector. In fact, there may be differences in waste production due to the business decisions made by farmers [80,81], including the quantity of olives milled on the farm or entrusted to third parties.

**Table 4:** Collinearity check related to the constructs and items included in the structural model.

Constructs	Personal Norms	Ascription of responsibility	Pro-environmental Behaviour
PN			1.364
AC	1.215	1.000	1.389
AR	1.215		
Variables			
Age			1.797
Education			1.234
Years of activity			1.164
Collaborators			1.757
Olive processed (t)			1.149
Type of milling activity			4.428
Wastes (mc)			4.489
Milling capacity (100 kg/h)			1.220
Costs (€/100 kg)			1.138

**Fig 2:**
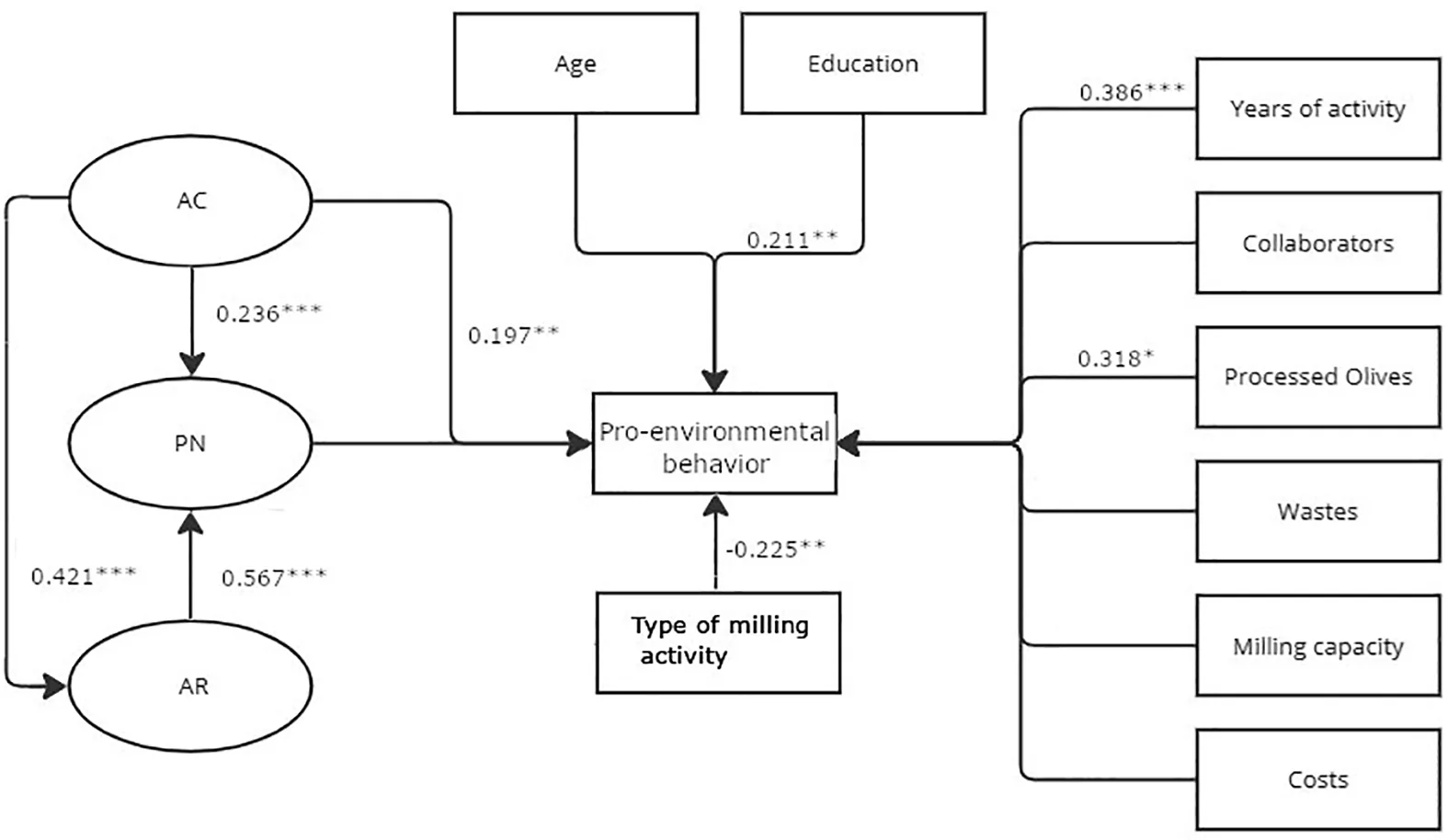
Graphic presentations of the relationships checked using the structural model. The results highlight the central role of AC and AR in shaping moral norms (PN) and the contribution of education, experience, and enterprise size to pro-environmental behaviour in the olive oil sector.

The relations of the structural model are graphically presented in Fig 2 and depicted quantitatively in Table 5. Several significant relationships were identified with a minimum p-value threshold of 0.1. This threshold was considered acceptable to identify statistically significant relationships, as p-values below 0.10 are commonly regarded as relevant in business and behavioural research contexts characterized by complex decision-making processes [75].

**Table 5:** Path coefficients and standard errors (SE) related to the structural model.

Constructs	Personal Norms (SE)	Ascription of responsibility (SE)	Pro-environmental behaviour (SE)
PN			−0.111 (0.102)
AC	0.236 (0.079)***	0.421 (0.082)***	0.197 (0.096)**
AR	0.567 (0.063)***		
Variables			
Age			−0.066 (0.096)
Education			0.211 (0.088)**
Years of activity			0.386 (0.095)***
Collaborators			−0.097 (0.078)
Olive processed (t)			0.318 (0.191)*
Type of milling activity			−0.225 (0.090)**
Wastes (mc)			−0.174 (0.175)
Milling capacity (100 kg/h)			−0.086 (0.087)
Costs (€/100 kg)			0.104 (0.099)
R square	0.481	0.170	0.221

p-values related to the path coefficients: * p < 0.1, ** p < 0.05, *** p < 0.01

Furthermore, the R^2^ values associated with the endogenous constructs and estimated through the structural equations indicate adequate explanatory power, in line with values reported in recent PLS-SEM studies [82,83].

Concerning H1, the empirical results provide only partial support for the overall adequacy of the NAM framework: although AC and AR significantly contribute to the activation of personal norms, PN themselves do not translate directly into observed pro-environmental behaviour. The detailed pattern underlying this partial validation is examined in the following paragraphs. The outcomes indicate a significant and positive association between Ascription of Responsibility (AR) and Personal Norms (PN), suggesting that individuals’ perceived environmental responsibility reinforces moral dimensions linked to sustainability and circularity within agricultural contexts [27]. Similarly, Awareness of Consequences (AC) exerts a significant influence on Personal Norms, suggesting that as entrepreneurs’ awareness of environmental issues increases, their environmentally oriented moral attitudes are strengthened. These outcomes confirm H2, showing that both AC and AR act as primary antecedents of moral obligation among millers. This pattern supports the central premise of the Norm Activation Model, which assumes that pro-environmental behaviour emerges when people both acknowledge the consequences of their actions (AC) and internalize personal responsibility (AR). In rural contexts, where environmental damage is often perceived as diffuse or collective, this cognitive-emotional link is essential to activate moral motivation, as highlighted in other studies [29,84].

Moreover, the observed strong relationship between AC and AR indicates the presence of a cascading effect within the NAM structure. In particular, heightened awareness of environmental impacts (AC) tends to amplify perceived responsibility (AR), thereby reinforcing personal moral norms (PN). This sequential dynamic clarifies how cognitive understanding of environmental issues can progressively evolve into a moral sense of obligation and, eventually, into behavioural readiness. In other words, awareness functions as the cognitive trigger that initiates the moral activation process, while responsibility serves as the emotional bridge linking awareness to personal norms [85,86]. This sequential dynamic refers to the empirical pathway AC → AR → PN observed in our model (H2), which is fully consistent with the original NAM logic [27,34] and with subsequent extensions of NAM to non-consumer decision settings [42], in which awareness of consequences activates ascription of responsibility, which in turn reinforces moral obligation.

Nonetheless, the analysis shows that PN does not significantly predict pro-environmental behaviour, thereby challenging the expectation that moral norms directly determine sustainable actions. This partial rejection of H1 underscores the enduring “attitude–behaviour gap” recorded in literature [84,87,88], wherein stated environmental values often fail to manifest in tangible behaviour. This pattern is also consistent with prior NAM-based work showing that personal norms tend to be weaker predictors of relatively costly or operationally demanding pro-environmental behaviours than they are of more general intentions [89].

In conventional agricultural settings like olive oil production, this gap may be intensified by factors such as short-term financial priorities, path-dependent production habits, and limited social interaction. As noted by Bamberg and Möser [90], situational constraints, including access to resources, community engagement, and peer influence, can moderate the strength of personal norms. In this context, the limited activation of personal norms among millers could be attributed to the persistence of intergenerational habits and the lack of structured support systems.

A particularly relevant finding emerging from the model concerns the direct effect of Awareness of Consequences (AC) on observed behaviour (β = 0.197, p < 0.05), in a context where Personal Norms (PN) do not show a significant direct effect. This pattern partially departs from the canonical Schwartz NAM, in which PN is expected to be the proximal driver of action, and AC and AR are supposed to operate only indirectly through the activation of personal norms. Rather than weakening the framework, this evidence suggests that NAM may need to be adapted when applied to small, family-run agri-food firms. In such contexts, AC appears to operate as a more directly action-oriented construct, because awareness of the environmental, sanitary and resource-related consequences of milling activities is closely tied to the operator’s sense of professional and managerial duty over the firm and its environmental footprint [14,30,37]. Olive oil millers who clearly perceive the negative externalities of their activity can translate this cognitive recognition into concrete CE practices without necessarily passing through more abstract moral norms, especially when normative activation is weakened by intergenerational habits, short-term economic pressures and limited peer interaction. This interpretation is consistent with prior empirical evidence on farmers, showing that moral and social concerns operate as direct determinants of pro-environmental production choices alongside, and sometimes in place of, purely economic considerations [40]. Conversely, the non-significant role of PN reinforces the well-known attitude–behaviour gap [89] and signals that, in this entrepreneurial context, generic moral norms are insufficient to overcome structural and economic barriers.

Nevertheless, given the significant influence of Awareness of Consequences on circular behaviour, the NAM framework remains a valuable tool for explaining part of the behavioural variability observed among millers.

Beyond the NAM constructs, the analysis identifies socio-demographic and structural characteristics as significant determinants of CE engagement, in line with prior research on psycho-social drivers of pro-environmental behaviour [26,39,90].

With regard to H3, the model confirms that socio-demographic characteristics significantly affect the adoption of CE practices among olive millers. Education level, in particular, shows a positive association with CE adoption [31; 40], suggesting that more educated entrepreneurs are more likely to recognise the long-term environmental and economic benefits of circular systems and to integrate sustainability into their managerial decision-making. Together with age, education contributes to a richer cognitive and informational endowment that lowers the perceived barriers to CE engagement [43,44]. Overall, these results confirm H3 and indicate that knowledge, environmental literacy and individual cognitive resources operate as relevant psycho-social predictors of pro-environmental behaviour in the olive milling sector.

Regarding H4, firm-level characteristics also emerge as significant determinants of CE adoption. Years of activity constitute a significant positive predictor: while previous research has shown that mere experience does not invariably translate into higher environmental engagement [91], accumulated technical competence and operational know-how can reduce cognitive barriers and strengthen perceived self-efficacy, thus supporting the uptake of pro-environmental practices [92,93]. Firm size, captured through the total quantity of olives processed as an indirect proxy [18], is likewise positively associated with CE adoption, in line with prior evidence showing that larger enterprises are generally better equipped to pursue sustainability innovations thanks to greater financial, technical and human capital, which collectively reduce perceived implementation risks [37,94,95]. It should be noted, however, that the empirical literature on the role of firm size in environmentally friendly farming decisions is not univocal: studies in different agri-food contexts have reported negative or non-significant effects of firm size on the adoption of cleaner agricultural practices [33], suggesting that this relationship is context-specific and may depend on sector characteristics, governance structures and the type of practice considered. A particularly informative result concerns the type of milling activity, which exerts a significant negative effect on CE adoption (β = −0.225, p < 0.01). The variable distinguishes between mills that process the operator’s own olives (within-farm milling, code 1) and mills that work mainly or exclusively on a sub-contracting basis, processing olives that belong to external producers (code 2). The negative coefficient therefore indicates that sub-contracting mills, i.e., those operating predominantly as service providers for third-party olive growers, are markedly less likely to adopt CE practices than vertically integrated farm-mills.

Several complementary explanations can be advanced for this result. In sub-contracting mills the by-products generated during extraction (pomace, olive mill wastewater, husks) are processed at the mill but remain legally and economically tied to multiple external clients, which weakens the mill's incentive to invest in dedicated valorisation pathways. The fragmentation of residue flows across many small client-producers further raises the transaction costs of any CE-oriented strategy [80,81]. By contrast, vertically integrated farm-mills manage the entire production process, from olive growing to bottling. This direct involvement increases their awareness of environmental consequences and their control over by-products, thus favouring CE adoption. The business model of sub-contracting mills is also more service-oriented, centred on milling volume and unit cost rather than on the long-term sustainability of the supply chain, which may further dampen their propensity to invest in circular solutions. These explanations are coherent with recent multi-case evidence on circular business models in the agri-food sector, which identifies supply-chain position, vertical integration and the configuration of residue ownership among the most critical factors for the valorisation of agricultural by-products [25]. These findings support H4 and indicate that firm-level structural conditions are critical co-determinants of CE adoption, complementing the moral activation captured by NAM, with sub-contracting mills emerging as a strategic but underserved node for the diffusion of CE practices.

The model offers empirical support for a hybrid explanatory framework, in which the internal motivations captured by NAM interact with external enabling factors, such as demographic and organizational attributes, to shape pro-environmental outcomes. H2, H3 and H4 are fully supported, while H1 is only partially validated, since personal norms alone do not translate into observable behaviour without enabling conditions.

These results are consistent with the literature on psycho-social drivers of pro-environmental behaviour [26,39,90,96], which conceptualizes sustainable action as a product of both individual cognition and social context. In the Sicilian olive sector, where small-scale and family-run enterprises dominate, this means that internal moral activation must be complemented by structural support systems, peer collaboration, and access to innovation resources.

## 5. Conclusion

### 5.1. Main outcomes

This study highlighted how the perception of environmental responsibility (Ascription of Responsibility) and awareness of environmental consequences (Awareness of Consequences) significantly affect the personal moral norms of olive oil millers, confirming the central role of these dimensions within the context of agricultural sustainability.

Indeed, the structural model suggests that the adoption of CE practices is influenced by both psychological and structural factors, although normative drivers do not directly translate into observable pro-environmental behaviour. These findings highlight a complex and non-linear link between moral intention and practical action within the sector.

Although normative dimensions remain important in shaping environmental responsibility, the implementation of CE practices in olive mills depends more strongly on operational, educational, and structural conditions. In particular, socio-demographic and firm-level characteristics, such as education level, work experience, company size, and type of milling activity, emerges as relevant predictors of the CE adoption, indicating that these factors substantially contribute to the behavioural variability observed among business owners.

### 5.2. Implications

The adoption of circular economy (CE) practices in olive mills can enhance the valorisation of by-products, thereby improving firm profitability. At the same time, environmentally friendly practices are increasingly associated with consumer preferences, which may translate into greater market recognition and higher product value. Overall, millers perceive CE as a promising pathway for sectoral development; however, its wider diffusion depends on strengthening both awareness and structural conditions.

From a policy perspective, this calls for multi-level interventions that combine informational campaigns, technical assistance, training, and financial incentives. The empirical results suggest that CE adoption should not rely solely on moral or normative appeals. Although moral responsibility contributes to shaping environmental awareness, actual implementation is more strongly driven by educational attainment and structural conditions, with Awareness of Consequences emerging as a key determinant. Enhancing producers’ understanding of the environmental, economic, and operational benefits of waste valorisation can therefore act as a central lever for accelerating adoption.

In this regard, practical instruments such as demonstration projects, pilot initiatives, and managerial training programs can help translate awareness into operational change by illustrating the feasibility and profitability of CE technologies. Given the role of education, interventions should extend beyond current entrepreneurs to include universities, agricultural schools, and vocational training systems that shape future sector skills.

Structural characteristics such as firm size and milling typology further indicate that the transition to CE requires adequate organisational and financial capacity. Entrepreneurial associations and sectoral networks can support this transition by facilitating knowledge exchange, collective investment, and advisory services, while CAP-related funds should be streamlined to reduce administrative barriers and better align with mills’ operational needs.

A more specific implication emerges with respect to milling typologies. Sub-contracting mills—processing olives for third-party producers—are significantly less likely to adopt CE practices than vertically integrated farm-mills. Policy interventions should therefore explicitly target the milling stage as a strategic node in the supply chain, not only olive producers. Measures may include training and technical assistance for mill operators, support for shared infrastructures to valorise by-products such as pomace, wastewater, and husks, and incentives for contractual arrangements that internalise by-product management responsibilities.

Accordingly, these findings also contribute to the CE literature by suggesting that, for agri-food entrepreneurs, the Norm Activation Model should be specified such that Awareness of Consequences (AC) operates not only indirectly through Ascription of Responsibility (AR) and Personal Norms (PN), but also directly on behaviour, particularly when the decision-maker coincides with the firm owner, thereby reinforcing the empirical evidence that awareness-building and structural support jointly act as key levers in translating environmental cognition into observable pro-environmental behaviour.

### 5.3. Limitations and future research

While this study provides useful information, certain limitations occurred and should be acknowledged to the readers. Firstly, the reliance on self-reported data could lead to bias, as the business owners may overstate or understate their engagement in CE practices due to social desirability or recall issues. Future prospective might therefore employ observational techniques or complementary data sources to better validate self-reported measures of behaviour.

Second, while this study highlights the importance of socio-demographic and structural characteristics in CE adoption, it does not fully account for the influence of external market dynamics, policy incentives, and financial constraints. Future research could examine how economic factors, regulatory frameworks, and access to funding shape the implementation of CE practices in agricultural systems.

Another limitation to consider is that the small sample size may lead to some instability in the estimates. However, this issue has been mitigated in this study by a sample size representing over 18% of the miller population, as well as by the use of a parsimonious model using PLS-SEM model, which is well suited to contexts with small populations.

Additionally, the study finds that the Norm Activation Model (NAM) partially explains pro-environmental behaviour regarding CE adoption. CE adoption was operationalised as a binary (dummy) outcome which may result in some loss of information but ensures greater parsimony of the model. To enrich this understanding, other behavioural frameworks, such as the Theory of Planned Behaviour (TPB) or the Value-Belief-Norm (VBN) theory, could be integrated with NAM to capture a broader array of psychological and motivational determinants. Future research could also extend this approach to additional agricultural sectors or conduct cross-country analyses to identify context-specific patterns and improve external validity.

Moreover, examining the role of networks, partnerships, and knowledge-sharing platforms in facilitating CE transitions would provide practical insights for strengthening collaboration among business owners, policymakers, and researchers.
